# Supplementary material for: Molecular Phylogenetic and Comparative Genomic Analysis of Pleurocordyceps fusiformispora sp. nov. and Perennicordyceps elaphomyceticola in the Family Polycephalomycetaceae
Source: J Fungi (Basel). 2024 Apr 19;10(4):297. doi: 10.3390/jof10040297 (PMC11050985; doi:10.3390/jof10040297)
Supplement: Supplementary file 1 [file jof-10-00297-s001.zip › jof-2945887-supplementary.pdf]

Supplementary materials for

Molecular Phylogenetic and Comparative Genomic Analysis of *Pleurocordyceps fusiformispora* sp. nov. and *Perennicordyceps elaphomyceticola* in the Family Polycephalomycetaceae

Zuoheng Liu [1,2,†], Yingling Lu [1,2,†], Dexiang Tang [1,2,†], Juye Zhu [1,2], Lijun Luo [1,2], Yue Chen [1,2] and Hong Yu [1,2,*]

[1] Yunnan Herbal Laboratory, College of Ecology and Environmental Sciences, Yunnan University, Kunming 650504, China; lzh1313159@163.com (Z.L.); lyinglingua@163.com (Y.L.); tangdx1516@163.com (D.T.); zhujuye@mail.ynu.edu.cn (J.Z.); yunllj07@163.com (L.L.); cy106daytoy@163.com (Y.C.)

[2] The International Joint Research Center for Sustainable Utilization of Cordyceps Bioresources in China and Southeast Asia, Yunnan University, Kunming 650091, China

* Correspondence: hongyu@ynu.edu.cn or herbfish@163.com; Tel.: +86-137-0067-6633

† These authors contributed equally to this work.

Table S1. Genomic assembly and functional annotation of *Pe. elaphomyceticola* and *Pl. fusiformispora* genome.

Item	Value		Item	Count		Percentage (%)	
	Pe. elaphomyceticola	***Pl. fusiformispora***		***Pe. elaphomyceticola***	***Pl. fusiformispora***	***Pe. elaphomyceticola***	***Pl. fusiformispora***
Total length (Mb)	31.51	33.02	NR	7,536	8,566	96.6898	97.2967
Max length(bp)	592,654	1,736,833	SwissProt	5,605	6,412	71.9143	72.8305

GC content (%)	48.79	50.08	KEGG	3,509	3,723	45.0218	42.2876
Total genes number	7,794	8,804	GO	5,302	6,119	68.0267	69.5025
Total genes length (bp)	12,410,008	14,320,079	EggNOg	7,196	8,183	92.3274	92.9464
Average gene length (bp)	1,592.2	1,626.5	P450	7,622	8,618	97.7932	97.8873
Gene Genome (%)	44.6288%	47.1169%	TCDB	1,334	1,520	17.1157	17.2649
Contigs	747	164					
Scaffolds	708	137					
Contigs N50	131,715	755,432					
Scaffolds N50	137,010	799,650					
Contigs N90	18,131	194,277					
Scaffolds N90	18,940	220,879					
Complete BUSCOs (%)	96.8%	97.6%					

Table S2. The biosynthesis gene cluster of putative secondary metabolites in two species of *Pe. elaphomceticola* and *Pl. fusiformispora*.

Species	BGCs	Location	From	To	Domain	Type	Most similar known cluster (%)
Pe. elaphomyceticola	Region 7.1	Scaffold 7.g25	74702	145697	A-A-A-P	NRPS	Unknown
	Region 9.1	Scaffold 9.g80	326610	364391	A-P-Te	NRPS	piperazine compound 1 piperazine compound 2 (50%)
	Region 13.1	Scaffold 13.g63	209865	243884	A-P-T	NRPS	**ε**-poly-lysine (100%)
	Region 14.1	Scaffold 14.g18	48607	93157	C-A-P-C	NRPS	Unknown
	Region 20.2	Scaffold 20.g33	127426	144511	C-A-P-A-C-A-P-C	NRPS	Unknown

Region 20.3	Scaffold 20.g36	149182	151779	C-A-P	NRPS	Unknown
Region 69.1	Scaffold 69.g5	1	42428	A-P-Te	NRPS	Ochrindole A (17%)
Region 79.1	Scaffold 79	1	43089		NRPS	Unknown
Region 82.1	Scaffold 82.g2	1	24294	A-P-Te	NRPS	Unknown
Region 93.3	Scaffold 93.g10	27990	30535	C-A	NRPS	Unknown
Region 125.1	Scaffold 125.g5	1	57332	C-A-C	NRPS	Unknown
Region 133.1	Scaffold 133.g6	1	51306	C-A	NRPS	Unknown
Region 143.1	Scaffold 143.g5	1	44723	P-A	NRPS	Unknown
Region 202.1	Scaffold 202.g1	1	28192	C-A-P-C	NRPS	Tubulysin A (6%)
Region 202.2	Scaffold 202.g2	14421	17416	A-P-C	NRPS	Tubulysin A (6%)
Region 42.1	Scaffold 42	561	47863		PKS,NRPS	Unknown
Region 61.1	Scaffold 61.g14	20743	65590	KS-AT-MT-KR-ACP-C-A-P-Te	PKS,NRPS	Triticone DABFC (57%)
Region 46.1	Scaffold 46.g23	71887	115252	KS-AT-PT-ACP-Te	NR-PKS	Viriditixin (55%)
Region 75.1	Scaffold 75.g27	56639	98993	SAT-KS-AT-PT-ACP	NR-PKS	Cryptosporioptide BAC (26%)
Region 93.2	Scaffold 93.g9	1579	50776	KS-AT	NR-PKS	Unknown
Region 206.1	Scaffold 206.g1	1	27003	KS-AT-DH	NR-PKS	Pyripyropene A (33%)
Region 133.2	Scaffold 133.g7	1	51306	KS-AT-DH-MT-KR-ACP	PR-PKS	Unknown

	Region 1.1	Scaffold 1.g23	77827	87337	KS-AT-MT-ER-KR-ACP	HR-PKS	Unknown
	Region 1.2	Scaffold1.g70	265444	310858	KS-AT-MT-ER-KR-ACP	HR-PKS	Unknown
	Region 14.2	Scaffold 14.g39	172672	214789	KS-AT-DH-MT-ER-KR-ACP	HR-PKS	LeucinostatinA B (10%)
	Region 20.1	Scaffold 20.g30	109233	171779	KS-AT-DH-MT-ER-KR-ACP	HR-PKS	Emericellamide AB (40%)
	Region 6.2	Scaffold 6	326876	348294		Terpene	Unknown
	Region 18.1	Scaffold 18	170706	192013		Terpene	Squalestatin S1 (40%)
	Region 26.1	Scaffold 26	75049	95531		Terpene	Unknown
	Region 28.1	Scaffold 28	117759	138919		Terpene	Unknown
	Region 139.1	Scaffold 139	20617	42485		Terpene	Unknown
	Region 2.1	Scaffold 2	232714	253931		Indole	Unknown
	Region 39.1	Scaffold 39	53070	117580		Indole,NRPS	Sirodesmin PL (33%)
	Region 6.1	Scaffold 6	216958	277296		Fungal-RIPP-like	Unknown
	Region 52.1	Scaffold 52	1	38481		Fungal-RIPP-like	Pyripyropene (33%)
	Region 120.1	Scaffold 120	8982	69323		Fungal-RIPP-like	Unknown
	Region 121.1	Scaffold 121	19846	70247		Fungal-RIPP-like	Unknown
	Region 134.1	Scaffold 134	1	62446		Fungal-RIPP-like,PKS	Ansaseomycin A B (13%)
	Region 93.1	Scaffold 93.g8	1579	50776	Te	Other	Fusaric acid (10%)
Pl.fusifor	Region 5.1	Scaffold 5.g64	411422	412973	A-P-C	NRPS	Unknown

mispora	Region 5.2	Scaffold 5.g65	414579	420793	C-A	NRPS	Unknown
	Region 6.2	Scaffold 6.g17	129742	132588	A-Te	NRPS	Unknown
	Region 6.8	Scaffold 6.g210	1135446	1138952	C-A	NRPS	Unknown
	Region 7.1	Scaffold 7	19894	64954		NRPS	Unknown
	Region 12.1	Scaffold 12	116619	170247		NRPS	Unknown
	Region 16.2	Scaffold 16.g53	335275	344607	A-A-C-P-C-P-C	NRPS	Unknown
	Region 19.4	Scaffold 19	341135	393626		NRPS	Unknown
	Region 21.4	Scaffold 21.g102	432651	435971	A-P-T	NRPS	**ε**-poly-lysine (100%)
	Region 23.1	Scaffold 23.g73	313835	315380	A	NRPS	Choline (100%)
	Region 26.1	Scaffold 26.g51	222968	225892	C-A	NRPS	Cyclopiazonic acid (42%)
	Region 28.1	Scaffold 28.g5	65124	68767	C-A-P	NRPS	Unknown
	Region 28.2	Scaffold 28.g7	90349	93806	C-A-P-C	NRPS	Unknown
	Region 4.2	Scaffold 4	617474	659177		NRPS-like	Unknown
	Region 12.2	Scaffold 12	497708	541193		NRPS-like	Unknown
	Region 46.1	Scaffold 46	46633	87271		NRPS-like	Unknown
	Region 5.3	Scaffold 5	1102121	1149442		PKS,NRPS	Unknown
	Region 14.2	Scaffold 14	634722	687260		PKS,NRPS	Ilicicolin H (62%)
	Region 19.3	Scaffold 19.g36	186594	192152	KR-ACP-C-A-P-Te	PKS,NRPS	Unknown
	Region 32.1	Scaffold 32.g2	45642	51144	KR-ACP-C-A-Te	PKS,NRPS	Lucilactaene (69%)
	Region 2.3	Scaffold 2	1393534	1442856		PKS,NRPS	Ascochlorin (50%)
	Region 2.2	Scaffold 2.g24	95089	97907	KS-AT	NR-PKS	Unknown
	Region 16.1	Scaffold 16.g49	315460	317847	PT-ACP-Te	NR-PKS	YWA1 (100%)
	Region 19.2	Scaffold 19.g35	180329	184881	KS-AT-DH-MT	NR-PKS	Tolypyridone (100%)
	Region 21.3	Scaffold 21.g67	308244	314240	SAT-KS-AT-PT-ACP-Te	NR-PKS	MonordenD/monocillinIV/ monocillin VII/ pochoninM/monocillin V/ monocillin II (100%)

Region 4.3	Scaffold 4.g306	1376326	1377484	KR-ACP	PR-PKS	Fusarin C (100%)
Region 41.1	Scaffold 41.g14	69586	77550	KS-AT-DH-MT-KR	PR-PKS	BetaenoneC/probetaenone I/ stemphyloxin II (28%)
Region 1.2	Scaffold 1.g291	1336070	1337484	ER-KR-ACP	HR-PKS	Unknown
Region 6.3	Scaffold 6.g30	193373	195963	ER-KR-ACP	HR-PKS	AKML BDAC (100%)
Region 6.5	Scaffold 6.g82	420764	429084	KS-AT-DH-MT-ER-KR-ACP	HR-PKS	Patulin (13%)
Region 6.6	Scaffold 6.g112	616842	624865	KS-AT-MT-ER-KR-ACP	HR-PKS	UNLL-YC2Q1O94PT (100%)
Region 6.7	Scaffold 6.g209	1125184	1133414	KS-AT-DH-MT-ER-KR-ACP	HR-PKS	Unknown
Region 7.2	Scaffold 7.g260	1109730	1116832	KS-AT-ER-KR-ACP	HR-PKS	Scytophycin (11%)
Region 14.1	Scaffold 14.g79	310982	314433	ER-KR-ACP	HR-PKS	Unknown
Region 21.2	Scaffold 21.g63	290083	297343	KS-AT-DH-ER-KR-ACP	HR-PKS	Monorden Dmonocillin IVmonocillin VIIpochonin Mmonocillin VmonocillinII (100%)
Region 68.1	Scaffold 68.g2	9458	17032	KS-AT-ER-KR-ACP	HR-PKS	4-epi-15-epi-brefeldin A (40%)
Region 1.3	Scaffold 1	1818360	1839982		Terpene	Unknown
Region 8.1	Scaffold 8	616055	637080		Terpene	Squalestatin S1 (40%)
Region 11.1	Scaffold 11	333198	354607		Terpene	Mangicol A (33%)
Region 21.1	Scaffold 21	169963	191672		Terpene	Unknown
Region 48.1	Scaffold 48	61502	82497		Terpene	Unknown
Region 19.1	Scaffold 19	26728	98386		NRPS,indole	Ergotamine (64%)
Region 4.1	Scaffold 4	470667	530852		Fungal-RIPP-like	Unknown

Region 6.4	Scaffold 6	266954	327797		Fungal-RIPP-like	Unknown
Region 15.1	Scaffold 15	202891	263124		Fungal-RIPP-like	Unknown
Region 1.1	Scaffold 1.g72	295273	298611	A-NOX-FNR	NRPS,Other	Unknown
Region 2.1	Scaffold 2.g23	90729	91739	P-Te	Other	Unknown
Region 6.1	Scaffold 6.g13	102660	105414	Caic-P-Te	Other	Unknown
Region 22.1	Scaffold 22.g66	317437	318249	Te	Other	Oxaleimide C (10%)
Region 24.1	Scaffold 24.g97	444447	445421	P	Other	Unknown

Table S3. Overview of biosynthetic gene clusters in the genomes of the two studied fungi.

	No of cluster	NRPS	PKS	NR-PKS	PR-PKS	HR-PKS	PKS, NRPS	Terpene	NRPS, Other	PKS, Other	Other
Pe. elaphomyceticola	39	15	9	4	1	4	2	5	1	1	6
Pl. fusiformispora	50	16	15	3	2	10	5	5	2		7

Table S4. Morphological comparison of asexual morph species of *Pleurocordyceps*.

Species	Host	Synnemata (mm)	Phialides (μm)	Conidia (μm)	References
Pl. agarica	*Ophiocordyceps* sp. or melolonthid larvae	Solitary, unbranched, agaricshaped, size (0.34–1.2×0.11–0.42); conidial mass pileus-like, light yellow to pale brown, size (0.08–0.25×0.36–0.99)	α-phialides lanceolate, length (30.7–81.9), base width (1.3–2.4), neck width (0.5–1.1); β-phialides narrowly lageniform or subulate, length (4.9–28.6), base width (1.8–3), neck width (0.4–0.8)	α-conidia globose to subglobose, size (2–3.1×1.8–2.9); β-conidia fusiform, catenate or clump together, size (3.8–6.8×1.7–3.2)	[25]
Pl. aurantiacus	Coleoptera larvae or *O. barnesii*	Emerging after 30 days, solitary or not solitary, branched or unbranched,	α-phialides, narrowly lageniform, 9.9–14.3 × 0.7–1.4. β-phialides, lanceolate or narrowly lageniform, 22.9–64.2 × 1–1.5	α-conidia, globose to subglobose, 1.8–2.2 × 1.4–1.9. β-conidia, fusiform, 3.2–3.9 × 1.4–1.8	[42]

		1.3–2.2 long, showing 1–2 radiating ringlike distributions			
Pl. lanceolatus	Lepidoptera larvae	0.2–1.2 cm long, 1–3 mm wide, lanceolate to corniform, solitary to crowded, stipitate, usually unbranched, rarely branched on the PDA, yellow to yellowish on the fresh specimen, covered with conidial masses, white on the PDA	*α*-phialides 12–18×1.3–1.6, directly from hyphae, solitary, usually unbranched, subulate, at the base, 8–14 long, tapering into a long neck, 2–4 long; β-phialides 20.2–28.9×1.6–2.5, branched into 2 or 3 phialides, narrowly lageniform to lanceolate	*α*-conidia 1.8–2.5, spherical, forming slimy conidial masses along the synnema; β-conidia 3–4.4×1.4–2.4, fusiform	[1]
Pl. marginaliradians	Cossidae larva	Emerging after 14 days, single or branched into 2 or 3 branched, 3.2–4.6 long, showing 1–2 radiating ringlike distributions	*α*-phialides, elongate lageniform, 11–14.4 × 1.2–1.8; β-phialides, 12.8–23.9 × 1.8–2.7, narrow slender to narrow lageniform	*α*-conidia 1.9–2.6 μm diam, globose, catenate, one-celled, pale yellow slimy in mass.β-conidia 3.1–3.9×1.6–2.1 μm fusiform, one-celled	[42]
Pl. parvicapitata	*Perennicordyceps elaphomyceticola*	Absent	Phialides 11.7–16.3×1.1–1.6, cylindrical at the base, 8–12 μm long, tapering into a long neck, 1–3.5 μm long	globose to subglobose, 2.8–3.6 × 2.4–3	[1]
Pl. sinensis	Lepidoptera larvae or *Ophiocordyceps sinensis*	Solitary, crowded, branched or unbranched, a length of up to 50–60 in culture, conidial mass yellow or yellow-orange	Lanceolate or narrowly lageniform, length (12.5–66), base width (1.4–3.5), neck width (0.6–1.8)	*α*-conidia, ovoid, 1.7–2.6 × 1.3–2; β-conidia, fusiform, 3.3–4.5 × 1.3–2	[32,60]

Pl. vitellina	*Ophiocordyceps nigrella*	Absent	α-phialides 12–37.7×1.5–2.7, hyaline, smooth, elongated lageniform, crowed, gathered in the middle of colony. β-phialides 11.6–28.7×1.9–3.1, hyaline, smooth, directly growing from hyphae, with or without metula at the base, solitary, lanceolate, ovate at the base, tapering into a short neck	α-conidia spherical, 2.5–3.2, one-celled, smooth-walled. β-conidia fusiform, 3.1–4.5×1.4–2.4, catenulate	[1]
Pl. yunnanensis	Hemiptera adults or *Ophio cordyceps nutans*	Solitary, caespitose or crowded, branched or unbranched, size (0.7–14×0.05–1.2); conidial mass white to yellow–brown, size (0.2–1.5×0.2–1.4)	α-phialides cylindrical to subulate, length (20.1–57.8), base width (1–2.3), neck width (0.5–1.3); β-phialides narrowly lageniform or subulate, length (7.1–30.6), base width (2.3–3.7), neck width (0.5–1.1)	α-conidia subglobose, ellipsoidal, size (1.4–2.5×1.2–2.2); β-conidia fusiform, catenate or clump together, size (2.8–5.7×1.1–2.7)	[24]
Pl. nutansis	*Ophiocordyceps nutans*	2–5 mm long, 0.1–0.4 mm wide, cylindrical, clavate, capitate, stipitate, crowded, simple, white to yellowish	Two types, both of the types observed on the same synnema. α-phialides 10–24×1–1.2, gathered at the apex of the synnema, arranged in a parallel palisade-like layer around the apex of the fertile head, hyaline, usually branched into 2–6 phialides, nar rowly slender lanceolate; β-phialides 10–14×1.1–1.8, solitary, scattered along the stipe, lanceolate, ovate at the base, 5–12 µm long, tapering into a long neck, 2–4 long	α-conidia 1.6–2.3, spherical, forming slimy conidial masses on the fertile head; β-conidia 3.5–5×1.3–2, fusiform, produced along stipe of the synnema	[1]
Pl. heilongtanensis	*Ophiocordyceps* sp.	3–10 long, 0.5–2 wide, scattered on the surface of host, cylindrical, stipitate, unbranched, white, with or without fertile head	α-phialides 6.8–25.3×1.2–2.8, hyaline, smooth, elongated lageniform, caespitose, palisade-like, crowed, gathered in the top of synnema, mostly branched into 2–4 phialides. β-phialides 9.6–19.5×1.2–3.1, hyaline, smooth, solitary, branched into 2 or 3 phialides, with or without metula at the base, directly growing from hyphae	α-conidia 2.3–3.1×1.3–1.9, subglobose to ovoid, in yellowish slimy mass. β-conidia 2.8–4.4×1.4–2.6 fusiform, one-celled	[1]

Pl. lianzhouensis	Lepidoptera larva or *Ophiocordyceps crinalis*	Unbranched or dichotomously branched, 10–20 tall, 0.5–1.5 wide, conidial mass not seen	In whorls or intercalary and terminal, terminally awlshaped, length (6–12), base width (1.3–1.7), neck width (0.5–0. 8)	Ellipsoidal, oblong to cylindrical, size (5–7×1.3–1.6)	[59]
Pl. fusiformispora	Lepidoptera larva or *Ophiocordyceps* sp.	Flaky, branched, 14–19 long, and 3–5 wide, showing radiating distributions.	α-phialides acropleurogenous on conidiophores, and solitary on hyphae; narrow lageniform or subulate, tapering abruptly from the base to the apex, 8.42–20.9 long, 1.3–2.9 wide at the base, and 0.6–1.6 wide at the apex. β-phialides solitary on hyphae, lanceolate, tapering gradually from the base to the apex, 8.7–14.8 long, 2.5–3.1 wide at the base, and 0.9–1.1 wide at the apex	α-conidia ovoid or elliptic, and occurring in the conidial mass on the agar or on the final portion of synnema, 2.9–4.8×1.3–3.1; β-conidia fusiform or long fusiform, and produced on the surface mycelium of colony, usually in chains on a phialide, 2.8–4.4×1.7–2.8	This study
